# Smartphone-Imaged HIV-1 Reverse-Transcription Loop-Mediated Isothermal Amplification (RT-LAMP) on a Chip from Whole Blood

**DOI:** 10.15302/J-ENG-2015072

**Published:** 2015-10-16

**Authors:** Gregory L. Damhorst, Carlos Duarte-Guevara, Weili Chen, Tanmay Ghonge, Brian T. Cunningham, Rashid Bashir

**Affiliations:** 1 Department of Bioengineering, The University of Illinois at Urbana-Champaign, Urbana, IL 61801, USA; 2 Micro and Nanotechnology Laboratory, The University of Illinois at Urbana-Champaign, Urbana, IL 61801, USA; 3 Department of Electrical and Computer Engineering, The University of Illinois at Urbana-Champaign, Urbana, IL 61801, USA

**Keywords:** human immunodeficiency virus (HIV), viral load, loop-mediated isothermal amplification, smartphone, point-of-care

## Abstract

Viral load measurements are an essential tool for the long-term clinical care of hum an immunodeficiency virus (HIV)-positive individuals. The gold standards in viral load instrumentation, however, are still too limited by their size, cost, and sophisticated operation for these measurements to be ubiquitous in remote settings with poor healthcare infrastructure, including parts of the world that are disproportionately affected by HIV infection. The challenge of developing a point-of-care platform capable of making viral load more accessible has been frequently approached but no solution has yet emerged that meets the practical requirements of low cost, portability, and ease-of-use. In this paper, we perform reverse-transcription loop-mediated isothermal amplification (RT-LAMP) on minimally processed HIV-spiked whole blood samples with a microfluidic and silicon microchip platform, and perform fluorescence measurements with a consumer smartphone. Our integrated assay shows amplification from as few as three viruses in a ~ 60 nL RT-LAMP droplet, corresponding to a whole blood concentration of 670 viruses per µL of whole blood. The technology contains greater power in a digital RT-LAMP approach that could be scaled up for the determination of viral load from a finger prick of blood in the clinical care of HIV-positive individuals. We demonstrate that all aspects of this viral load approach, from a drop of blood to imaging the RT-LAMP reaction, are compatible with lab-on-a-chip components and mobile instrumentation.

## 1 Introduction

Human immunodeficiency virus (HIV) affects 36.9 million people worldwide [1]. During the course of nearly four decades since the emergence of HIV on a pandemic scale, advances in antiretroviral therapy have transformed HIV infection from a death sentence into a chronic illness that can have little impact on life expectancy for those in whom the infection is properly managed [2]. At the population level, rates of new infections, mother-to-child transmission, and deaths from HIV-related causes are declining [2]. However, the lack of availability of the appropriate diagnostic technologies essential to informing treatment in routine HIV care is still among the chief barriers preventing access to the standard of care for millions of HIV-positive individuals worldwide, particularly in resource-limited settings.

CD4+ T lymphocyte counts and blood plasma viral load are the two core diagnostic measurements broadly considered essential to HIV care, as they both guide the initiation of therapy and indicate the efficacy of each individual’s treatment regimen [3]. CD4 counts, traditionally performed by flow cytometry, are increasingly available in remote settings due to the introduction of new portable platforms [4–7]. Viral load platforms, on the other hand, are well behind CD4 technologies in penetrating the developing world. Viral load instruments are traditionally reverse-transcription polymerase chain reaction (RT-PCR), nucleic acid sequence-based amplification (NASBA), or branched DNA (bDNA) assays; although these can be capable of detecting fewer than ten viral RNA copies per mL of blood plasma, these instruments require a laboratory setting, extensive sample handling, and sophisticated processing [6, 8–10].

One promising solution that can help address this issue is loop-mediated isothermal amplification (LAMP) [11]. LAMP emerged in the early 2000s as an alternative to PCR for nucleic acid detection [12]. LAMP is attractive for point-of-care applications because, unlike PCR, it does not require temperature cycling (isothermal at 60–65 °C), and because it is typically less sensitive than PCR to amplification inhibitors. RT-LAMP assays for HIV were first described soon after the introduction of the initial concept [13, 14], and there have been several reports since then regarding variations of the assay, including efforts toward point-of-care applications [15– 21]. Among the novel LAMP approaches applied to HIV that are presented in these reports are a battery-powered handheld microfluidic system that was demonstrated with purified DNA [20], a SlipChip device for digital LAMP [21], and an electricity-free heating container that facilitates a qualitative RT-LAMP assay on minimally processed whole blood [17, 18]. To date, however, an approach capable of performing a quantitative RT-LAMP assay from a drop of whole blood on a platform compatible with a fully automated, portable device has not been presented.

Traditionally, it is believed that nucleic acid amplification requires complete purification of the RNA or DNA target in order to be compatible with the amplification reaction. The robustness of LAMP, however, has disrupted this thinking. Whole blood treated only with a cell lysis buffer has been employed by Curtis et al. for HIV LAMP in a qualitative measurement in an electricity-free heating device [17]. We present here RT-LAMP with minimally processed lysed whole blood for a quantitative measurement of HIV viral load capable of detecting as few as three whole virus particles per ~ 60 nL reaction droplet.

Our approach to RT-LAMP HIV viral load measurements begins with a drop of whole blood. The data presented here demonstrate the potential of this approach to be developed into a fully automated mobile device that does not require manual processing. First, we compare and contrast the performance of the RT-LAMP reaction on a standard laboratory thermocycler, both with purified viral RNA in water and with whole virus particles in whole blood that has only been treated with a cell lysis buffer. Next, we implement a simple microfluidic mixing module in order to show that the whole blood lysis step can be performed on a chip without the loss of analyte or interference with the detection assay. We then move to a microchip platform and characterize the RT-LAMP reaction with purified RNA and lysed whole blood spiked with viral RNA, imaging with both a standard fluorescence microscope and a consumer smartphone without hardware modifications in order to compare and contrast standard and novel techniques. To demonstrate the robustness of the assay, we show the compatibility of the on-chip reaction with the presence of viral RNA from the hepatitis C virus (HCV) and viral DNA from the hepatitis B virus (HBV), which do not cross-react. We then combine the microfluidic lysis, microchip reaction platform, and smartphone imaging in order to demonstrate the capability of our platform to quantitatively determine HIV viral load from a drop of blood. Finally, we discuss merits and drawbacks, as well as the potential for this approach to address the need for point-of-care viral load technology.

## 2 Materials and methods

### 2.1 Samples

#### Whole blood

Whole venous blood samples were drawn from HIV-negative donors with a syringe and transferred to 4 mL BD Va cutainer K2 EDTA collection tubes. Tubes were stored at room temperature on a sample rotisserie until used for experiments.

#### Viruses

HIV-1, strain IIIB propagated in the H9 human T lymphocyte cell line was purchased from Advanced Biotech nologies, Inc. Virus stock was provided in purified form at a concentration of 6.7E+10 vp· mL^−1^ (vp is short for virus particles) in storage buffer containing 10 mmol· L^−1^ Tris, 150 mmol·L^−1^ NaCl, and 1 mmol·L^−1^ ethylene diamine tetraacetic acid (EDTA) at a pH of 7.5. Viruses used for experiments in whole particle form were diluted from aliquots of the stock, either in additional storage buffer prepared in-house or in phosphate buffered saline (PBS) from Fisher Scientific.

#### Viral nucleic acids

Synthetic HBV DNA (ATCC® VR-3232SD™) and synthetic HCV RNA (ATCC® VR-3233SD™) were purchased from American Type Culture Collection (ATCC). HIV-1 RNA was purified from HIV-1 IIIB whole particles using the PureLink® Viral RNA/DNA Mini Kit from Life Technologies. Two methods were used to produce dilutions of HIV-1 RNA for thermocycler characterization of the RT-LAMP reaction with purified RNA. In method 1, whole virus particles were diluted in PBS and each dilution was separately purified in the PureLink® kit. In method 2, 10 µL of 6.7E+10 vp· mL^−1^ was added to 190 µL PBS to meet kit specifications, and was then purified and eluted in 150 µL RNase-free water for a final concentration of 4.47E+9 vp·mL^−1^ (or 8.93E+9 RNA copies per mL). This purified RNA was then aliquoted and stored at −80 °C until use.

Microchip RT-LAMP experiments were performed with this viral RNA, with the exception of the integrated experiment. The experiments performed with purified RNA in water were done because purified RNA is the standard analyte in reverse-transcription nucleic acid amplification assays and these experiments serve as a basis for comparison to lysed whole blood. Whole virus particles are the ideal analyte in whole blood; however, the preliminary microchip experiments were performed with viral RNA spiked in whole blood for biosafety reasons while the technique was being developed. Preliminary “macroscale” amplification experiments with whole blood in a thermocycler (not on a microchip) did include whole virus particles because the technique was compatible with biosafety practices. To perform the final, integrated on-chip experiment, an apparatus was constructed in a biosafety cabinet in order to accommodate microchip experiments with whole virus particles.

#### Blood cell lysis

The whole blood lysis buffer was based on the work by Curtis et al. [17] and contained 2.5 mmol· L^−1^ KHCO_3_, 37.5 mmol·L^−1^ NH_4_Cl, and 0.025 mmol·L^−1^ EDTA. A 1:4 ratio of blood to lysis buffer was used for all lysed blood experiments. In preliminary experiments, blood and lysis buffer were metered and mixed with a manual pipettor, while the final integrated experiment and the preliminary experiment to characterize microfluidic mixing employed on-chip lysis in a microfluidic channel. For microfluidic mixing, the ratio of volumes mixed was fixed to 1:4 by setting relative flow rates from two syringe pumps driving each component.

### 2.2 RT-LAMP

#### Reaction components

The RT-LAMP assay was adapted from the work by Curtis et al. [17]. Reaction concentrations of buffers were 1× isothermal amplification buffer, 1.4m mol·L ^−1^ deoxyribonucleoside triphosphates (dNTPs), and 10 mmol·L^−1^ MgSO_4_ from New England Biolabs, and 0.4 mol ·L ^−1^ betaine from Sigma-Aldrich. In some cases, where indicated, 0.8 mol·L^−1^ betaine was used. These reaction buffer components were prepared in appropriate ratios in bulk and stored at −20 °C between experiments. Enzymes and DNA intercalating dye were added separately to this buffer mix for a complete master mix that was freshly prepared for each experiment. The RT-LAMP enzymes used in the reaction were 0.64 U· µL ^−1^*Bst* 2.0 DNA polymerase and 0.08 U· µL^−1^ AMV reverse transcriptase fro m New Engl and Biolabs. 1× EvaGreen from Biotium, a double-stranded DNA (dsDNA) intercalating dye, was included in the reaction for the detection of reaction products.

#### Primers

Six LAMP primers were based on the study by Curtis et al. [17], including a six-primer set containing 0.2 µmol· L^−1^ each of F3 (5’-AGTTCCCTTAGATAAAGACTT-3’) and B3 ( 5’- CCTACATACAAATCATCCATGT- 3’) primers, 1.6 µmol· L ^−1^ each of forward inner primer (FIP) (5’-GTGGAAGCACATT GTACTGATATCTTTTTGGAAGTATACTGCAT-TTACCAT-3’) and backward inner primer (BIP) (5’-GGAAAGGATCACCAGCAATATTCCTCTGGATTTTGTTTTCTAAAAGGC-3’), and 0.8 µmol·L^−1^ each of LoopF (5’-GGTGTCTCATTGTT TATACTA-3’) and LoopB (5’-GCATGACA-AAAATCTTA GA-3’) primers.

#### Negative controls

All amplification experiments, whether in the thermocycler or on the microchip, included negative controls that consisted either of water without RNA or lysed blood without viruses/viral RNA, according to the nature of the positive samples being tested. Amplification of the negative control within the reaction timeframe was considered to be an indication of a contaminated test. Fluorescence curves are not presented for these negative controls, although they were included in every experiment.

#### Reaction platforms

RT-LAMP reactions were performed on two different platforms at various stages of this study. For the purpose of establishing the RT-LAMP reaction and comparing and contrasting purified RNA in water with lysed whole blood, standard 25 µL reactions were performed in 0.2 mL reaction tubes in an Eppendorf Mastercycler® ep realplex Real-Time PCR System. The thermocycler was also used for RT-LAMP reactions characterizing the microfluidic mixing module in order to eliminate possible noise introduced by the microchip system.

To develop the microchip amplification, several microchip experiments were performed, beginning with RNA in water and RNA-spiked lysed whole blood (RNA was used for bio-safety reasons in these preliminary experiments as explained above). Each individual droplet (reaction) on the microchip contained approximately 60 nL and the entire microchip was placed in a copper bowl, as described below, and heated on an INSTEC STC200 heating stage. Imaging was initially performed with a Nikon Eclipse FN1 fluorescence microscope in order to employ a standard imaging method. Later, a Samsung Galaxy Note 4 smartphone was introduced. Both the fluorescence microscope and the smartphone were used in order to compare the imaging capabilities of the smartphone with those of standard laboratory imaging equipment. The reactions were initially incubated at 60 °C in the commercial thermocycler and later at 65 °C for the on-chip experiments. Fluorescence measurements were performed every 60 s with the thermocycler and fluorescence microscope, but increased to every 30 s with the smartphone platform.

Data are presented in this paper for microchip reactions with purified RNA in water imaged with a microscope and RNA-spiked lysed whole blood imaged with a smartphone. The intermediate experiment, RNA-spiked lysed whole blood reactions imaged with a microscope, is provided in the Supplementary Information.

### 2.3 Microfluidic lysis module

#### Fabrication

The microfluidic lysis module is based on an earlier design that was reported previously [22]. The polydimethylsiloxane (PDMS)-on-glass microfluidic channel was made from an SU-8 master mold fabricated using standard clean-room photolithography techniques. Uncured PDMS was poured over the SU-8 master, degassed in a desiccator, and cured on a hot plate at 60 °C. Holes for tubing connections were punched into the PDMS with a 1.5 mm biopsy punch, prior to solvent degreasing and the oxygen plasma surface activation of both the PDMS and a glass microscope slide in a Diener PICO plasma system. Activated surfaces of the PDMS and glass were brought into contact following surface activation and heated at 60–70 °C on a hot plate, producing covalent bonds between the two pieces.

#### Fluidic apparatus

The microfluidic lysis experiments involving whole HIV particles were performed with a fluidic apparatus that interfaced with the PDMS microfluidic chips and consisted of syringe pumps and high-performance liquid chromatography (HPLC) valves built within a biosafety cabinet. Biosafety level 2+ protocols were followed.

### 2.4 On-chip RT-LAMP

#### Chip fabrication

Microchip RT-LAMP experiments employed a microfabricated silicon substrate [23]. Briefly, a silicon wafer was thermally oxidized to create a silicon oxide layer of ~ 150 nm. The oxide was then patterned with photolithography and a hydrofluoric acid etch step, exposing the silicon where the wells would be etched. The wafer was then immersed in a heated tetramethylammonium hydroxide (TMAH) bath for 18 h in order to anisotropically etch the silicon, creating inverted square pyramids that would later be used as reaction wells. An approximation of the dimensions of the inverted square pyramids is provided in the Supplementary Information.

#### Chip preparation

Chips for all microchip RT-LAMP experiments were prepared in the following manner: First, the microchip was cleaned in a piranha solution containing 1:3 30% hydrogen peroxide and sulfuric acid for 10 min and then it was rinsed in deionized water. Each chip was then degreased with acetone, methanol, and isopropanol and dried by blowing with nitrogen gas. To produce a hydrophobic surface to promote stability of droplets, the chip was rinsed with Sigmacote® from Sigma-Aldrich by pipetting the solution repeatedly over the surface of the chip. The chip was then rinsed briefly with isopropanol, dried by blowing with nitrogen gas, and placed in a copper bowl.

#### Microinjection

A Narishige IM-300 Microinjector with Eppendorf VacuTip microinjection holding capillary (15 µm in inner diameter, 100 µm in outer diameter) was used both to spot primers and to place reaction droplets. A 20 ms injection pulse was used, resulting in a droplet of approximately 60 nL. The microinjection procedure was performed after chip cleaning and preparation as follows: LAMP DNA primers in Tris-EDTA (TE) buffer were diluted in water to the final reaction concentration. Droplets were placed in all 36 wells of the microchip array using the microinjection system and a 3D micromanipulator (MCL-D331) from World Precision Systems. The process was visualized with a Leica MZFLIII microscope. Droplets containing primers were allowed to dry completely, leaving dehydrated DNA LAMP primers in the reaction wells. Following visual confirmation that all droplets had dehydrated, the chip was submerged in heavy mineral oil (Fisher) and placed in a desiccator to remove air bubbles. The primary function of the mineral oil was to protect the reaction droplets from evaporation during heating at 65 °C.

During degassing, the primer-less RT-LAMP reaction was prepared and transferred to the microinjection capillary. Reaction droplets were then placed in each well by lightly contacting the bottom of each reaction well, injecting a droplet of approximately 60 nL, and lifting the capillary out of the oil. The chip containing all 36 droplets submerged in oil in the copper bowl was then transferred to the heating stage and imaging apparatus (fluorescence microscope or smartphone apparatus).

Primer spotting and reaction droplet placement for the integrated experiment were performed in the same manner but within a biosafety cabinet under a Leica EZ4D microscope with a built-in camera providing a live video feed to a personal computer (PC).

### 2.5 Fluorescence microscopy

Fluorescence microscopy images were captured with a Nikon Eclipse FN1 fluorescence microscope with a 2× objective and a Nikon 96311 B-2E/C FITC fluorescence filter. NIS Elements software was used to capture fluorescence images for RT-LAMP reactions containing purified RNA in water with 6.3× gain and 1 s exposure time. Additional measurements (presented in the Supplementary Information) with lysed whole blood spiked with viral RNA imaged with the microscope required 8× gain and a 2 s exposure time to compensate for decreased overall fluorescence intensity.

### 2.6 Smartphone imaging

#### Apparatus

**Figure 1:**
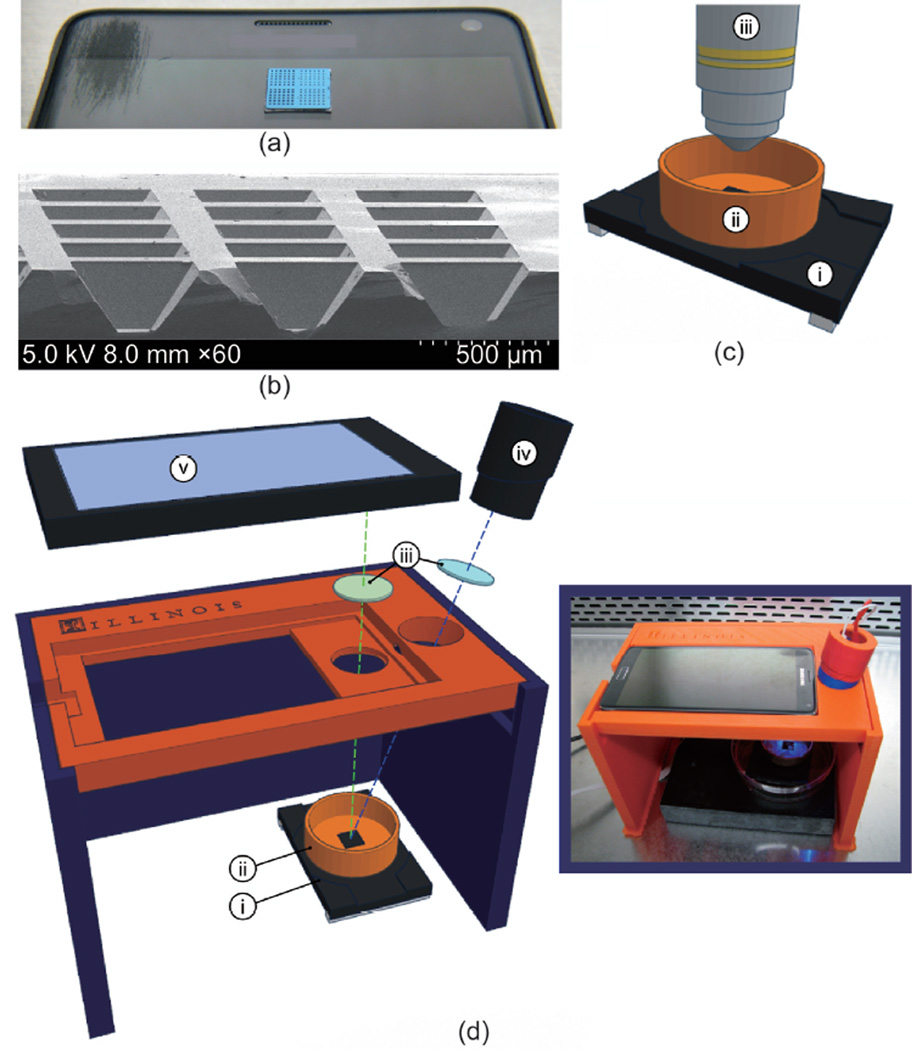
The RT-LAMP substrate and smartphone apparatus (a) Image of 1 cm × 1 cm silicon microchip substrate sitting on a Samsung smartphone. (b) Scanning electron microscopy cross-section of 160 µm-deep reaction wells. (c) Schematic of microchip and heating stage in fluorescence microscope apparatus, including: (i) heating stage, (ii) copper base containing mineral oil, and (iii) fluorescence microscope objective. (d) Expanded diagram of smartphone LAMP apparatus, including: (i) heating stage, (ii) copper base containing mineral oil, (iii) wavelength filters placed in front of the LED and smartphone camera, (iv) blue LED light source, and (v) smartphone. (d-inset) Image of apparatus assembled in biosafety cabinet.

A Samsung Galaxy Note 4 smartphone was purchased for the imaging of the RT-LAMP reaction on the microchip substrate. The smartphone hardware was not modified from its factory conditions. A Thorlabs 530 nm Longpass Colored Glass Filter was placed between the camera and the chip to isolate the fluorophore emission wavelengths. A 3D-printed cradle (Figure 1) was designed to position the smart-phone horizontally with the camera directly above the microchip. A mounting cylinder was also 3D printed to hold an Opto Diode Corp high-output blue light-emitting diode (LED) and a Thorlabs Shortpass Filter with a 500 nm cut-off wavelength, which fit within the cradle and illuminated the microchip from an angle. The LED was powered with 3 V from an Agilent E364xA DC power supply with an automated on/off function controlled with a MATLAB script. It was also determined that the blue LED could be adequately powered by a standard 3 V lithium coin battery, but the DC power supply was used for the purpose of PC control.

#### Software

Due to biosafety considerations, the entire smart-phone imaging apparatus was placed inside a biosafety cabinet when performing integrated measurements. For this reason, remote control of the imaging function was desired, so the Android application IP Webcam [24] was downloaded from the Google Play store and installed on the smartphone. This application transmits a live image over the network, which can be viewed in real time in a web browser. The browser interface allowed for control of the smartphone camera’s focus, exposure, and gain. Imaging of the RT-LAMP reaction was performed with the following parameters set in the IP Webcam web browser interface: 8× zoom, 99% stream quality, exposure compensation of 4, and “night vision” function with a gain of 10× and exposure 10.

A MATLAB script was written to automate the image-capture process. The script was initialized simultaneously with the activation of the heating stage. The MATLAB script imaged the reaction in the following sequence: Switch on blue LED, delay 3 s; capture image from IP Webcam web browser interface, delay 2 s; and finally switch off blue LED. This process was repeated every 30 s while each reaction was imaged.

### 2.7 Data analysis

#### Image analysis

Images recorded with fluorescence microscopy or the smart-phone imaging apparatus were saved as TIFF (microscope) or JPEG (smartphone) files and fluorescence intensity was analyzed. For this analysis, the physical location of each droplet was identified manually in a MATLAB script by importing and displaying the image in a MATLAB figure and adjusting the position of square boxes outlining each droplet. Grayscale TIFF images were imported as a matrix of 16-bit unsigned integers (range 0–65 535) representing each pixel in the image. Grayscale JPEG images were imported as an array of 8-bit unsigned integers (range 0–255) representing each pixel in the image.

Following manual identification of droplet positions, a MATLAB script automated the analysis of each droplet by averaging the numerical value of all pixels within the region defined by the box outlining each droplet. Absolute numerical values are a function of the range of integer values (8-bit or 16-bit), as well as the exposure time and gain of the camera, ambient light in the laboratory, and other factors. For this reason, the baseline is subtracted from each measurement as described below, and fluorescence measurements in this paper are presented in arbitrary units (AU).

#### Threshold time analysis

Threshold time was determined from raw fluorescence data on all platforms. First, the baseline was removed by subtracting an early fluorescence measurement from all subsequent measurements. For all thermocycler measurements and the microscope measurements with purified RNA in water, this was the first fluorescence value or image recorded. For smartphone measurements, the auto fluorescence of whole blood at room temperature was observed to decrease quickly upon initial heating of the chip. Thus, the baseline was defined to be 90 s after initialization of the heating, or the third image recorded by the smartphone.

The threshold time for each individual reaction was approximated from baseline-subtracted fluorescence curves by determining the measurement *n* at which the signal exceeded 20% of the maximum fluorescence value it achieved during the course of the entire measurement. After determining *n*, a linear fit was determined by the fluorescence values *I_n_* and *I*_*n*−1_ in the form *I*(*x*) = *mx* + *b* and the threshold time *T*_t_ = (0.2 × *I*_max_ − *b*)/*m* was determined.

## 3 Results

### 3.1 Characterization of RT-LAMP in a benchtop thermocycler

#### Purified viral RNA in water

**Figure 2:**
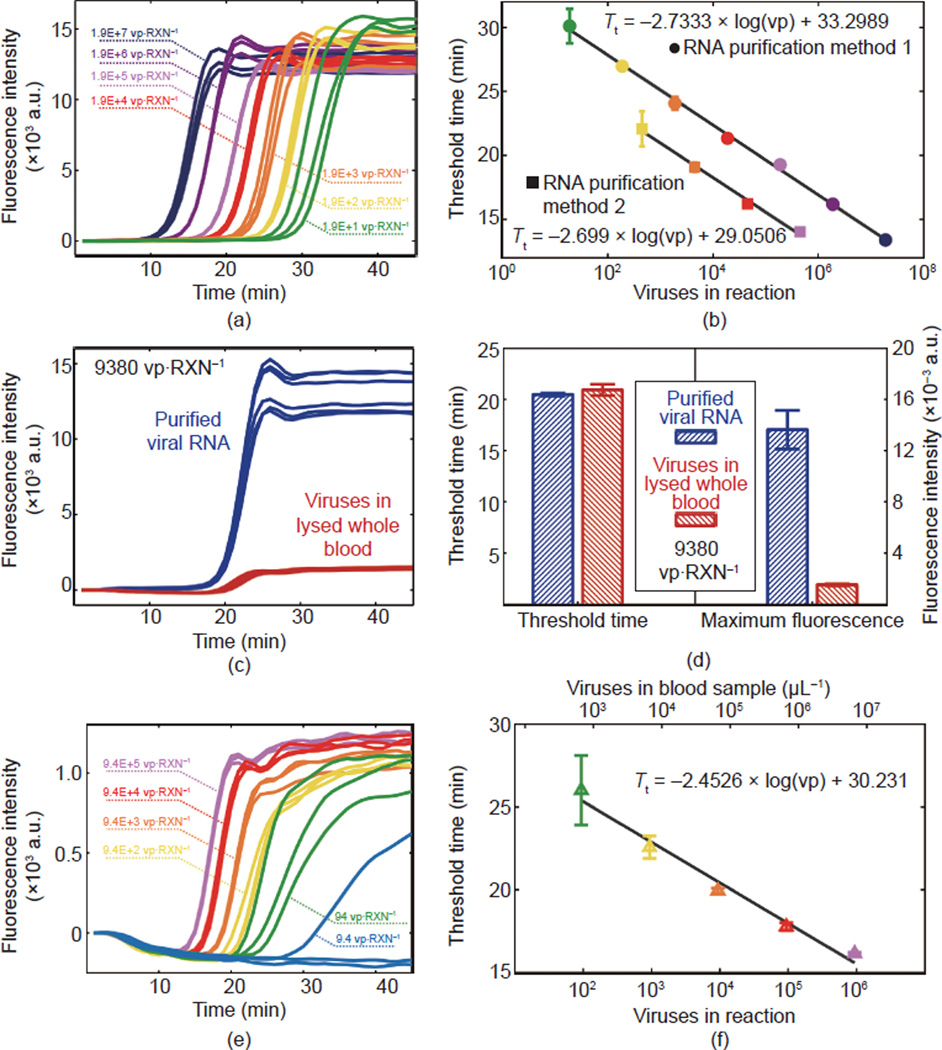
RT-LAMP performed in a standard benchtop thermocycler (a) Raw fluorescence data for RT-LAMP of viral RNA diluted and purified from dilutions of whole HIV-1 IIIB virus particles (RNA purification method 1). (b) Threshold time curves determined by calculating the time at which fluorescence curves exceed 20% of their maximum value. Data are included for both methods of producing purified viral RNA. (c) Fluorescence curves from six replicates of each condition comparing RT-LAMP in virus-spiked whole blood versus purified RNA. All reactions contained the equivalent of 9380 virus particles. (d) Comparisons of threshold time and overall fluorescence intensity for both conditions. (e) Fluorescence curves and (f) standard curve for RT-LAMP with HIV-1 IIIB whole viruses spiked in whole blood. The 9.4 vp·RXN^−1^ sample is not included in (f) due to inconsistent amplification of all replicates. RXN is short for reaction.

The first experiment, presented in Figure 2(a) and (b), was performed in order to characterize the RT-LAMP reaction with purified analyte in a standard thermocycler apparatus. HIV-1 IIIB RNA was purified by two different methods as described in Section 2.1. RT-LAMP fluorescence curves for the first method are shown in Figure 2(a), and the calculated threshold time versus the average number of viruses in each reaction is shown in Figure 2(b). Linear fits to threshold time versus log of viruses showed a difference in slope of less than 1.3% but a vertical offset of more than 4 min in the *y*-intercept.

#### Comparison with lysed whole blood

The next experiment compared the threshold time and fluorescence intensity of RT-LAMP containing 9380 whole virus particles of HIV-1 IIIB in lysed blood to the corresponding amount of purified HIV-1 RNA in water as an initial test of the feasibility of RT-LAMP in lysed whole blood. Figure 2(c) shows fluorescence measurements and Figure 2(d) provides bar charts comparing threshold time and maximum fluorescence value. Six replicates were performed of each of the two conditions, and the average threshold time in lysed blood varied by less than 2.3% compared to RNA in water, and gave a *P* of 0.0755 in a standard two-sample *t* test. The maximum overall fluorescence intensity (determined from raw fluorescence measurements with the baseline subtracted) showed a decrease in fluorescence signal of 88.93% in lysed whole blood compared to purified RNA in water.

#### Standard curve in lysed whole blood

An experiment with whole virus particles at a range of concentrations was then performed in order to characterize the RT-LAMP reaction on an ideal platform but with a minimally processed sample. Figure 2(e) (fluorescence intensity curves) and (f) (threshold time versus virus number) show that even with a ten-fold reduced overall fluorescence intensity, amplification curves could still be observed and threshold times analyzed. A linear fit to the threshold time versus the log of virus number gives a slope comparable to purified RNA curves (10.3% difference compared to method 1 and 9.1% difference compared to method 2 in Figure 2(b)). Due to inconsistent amplification of all replicates, the 9.4 vp·RXN^−1^ sample is not included in the threshold time curve. Further characterization of the lysed whole blood RT-LAMP reaction was performed by examining variations on the ratio of blood sample to lysis buffer. Results are provided in the Supplementary Information.

### 3.2 Microfluidic blood lysis module

**Figure 3:**
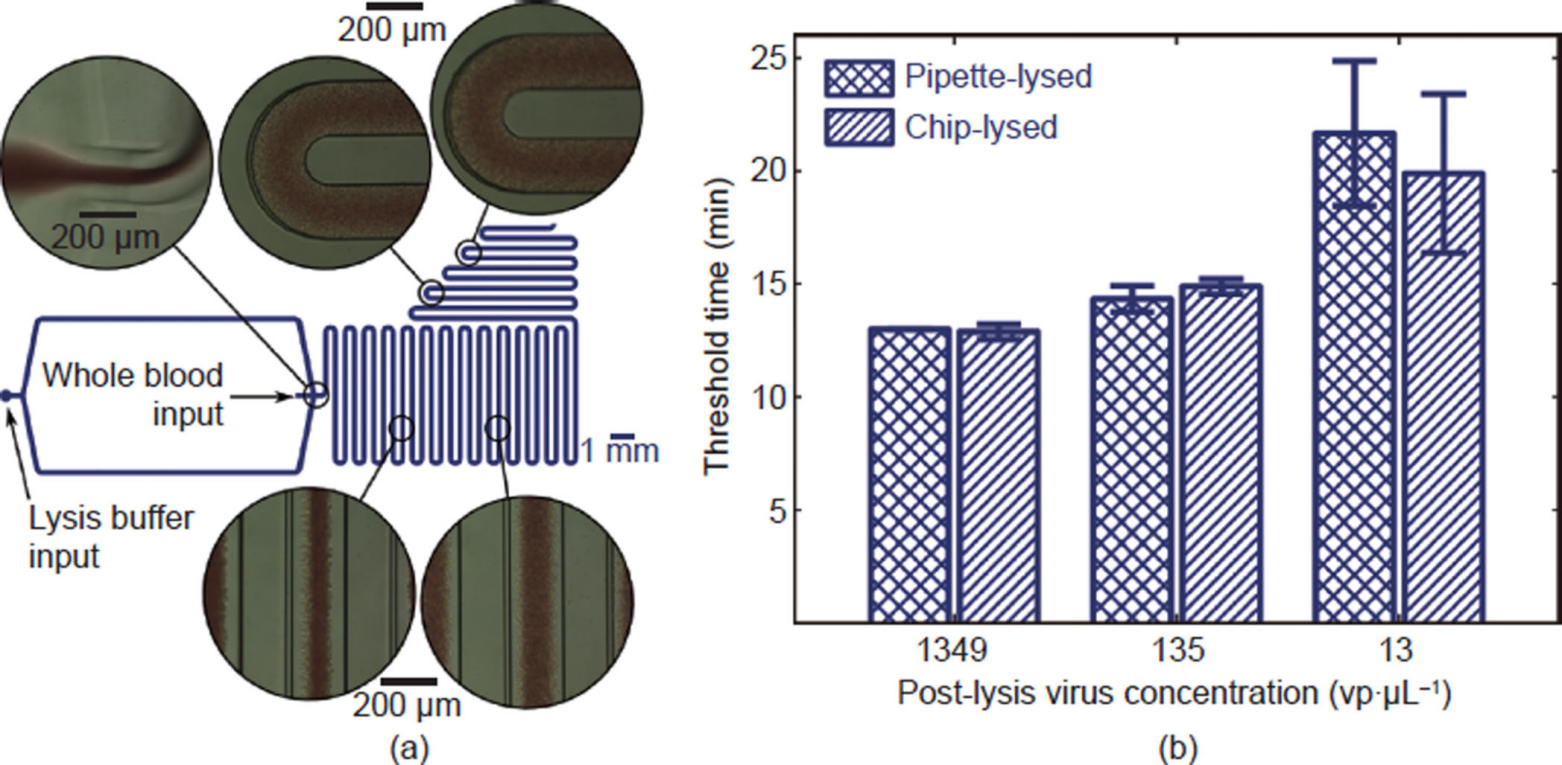
Microfluidic lysis of whole blood samples (a) Diagram of the microfluidic device with bright-field microscopy at various points in the channel with their approximate locations indicated. (b) A comparison of threshold time at three virus concentrations for chip-lysed versus pipette-lysed samples containing whole virus particles. These data verify that the microfluidic lysis method does not result in significant differences in signal compared to the manual method.

On-chip lysis in a microfluidic channel, shown in Figure 3(a), was independently characterized in order to determine the potential for automated sample handling and to characterize any impact of the microfluidic mixing on the overall method. Whole blood samples spiked with three different concentrations of HIV-1 IIIB were each mixed with lysis buffer in the polydimethylsiloxane (PDMS) mixing chip driven by the fluidics apparatus in a biosafety cabinet, as described in Section 2.3. Output from the microfluidic chip was collected on three separate instances from each sample and analyzed separately with RT-LAMP in a thermocycler. Triplicate RT-LAMP reactions were performed for each of the three collections, resulting in nine total RT-LAMP reactions for each virus concentration investigated. As a control, the same spiked blood samples were added to lysis buffer, mixed with a pipette, and analyzed with RT-LAMP in triplicate (i.e., three control reactions in total for each virus concentration). Figure 3(b) shows the results. Mean threshold times differed from those of the manually-pipetted control by 0.85%, 3.88%, and 8.21% for post-lysis virus concentrations of 1349 vp· µL^−1^, 135 vp·µL^−1^, and 13 vp·µL^−1^, respectively.

### 3.3 On-chip RT-LAMP

**Figure 4:**
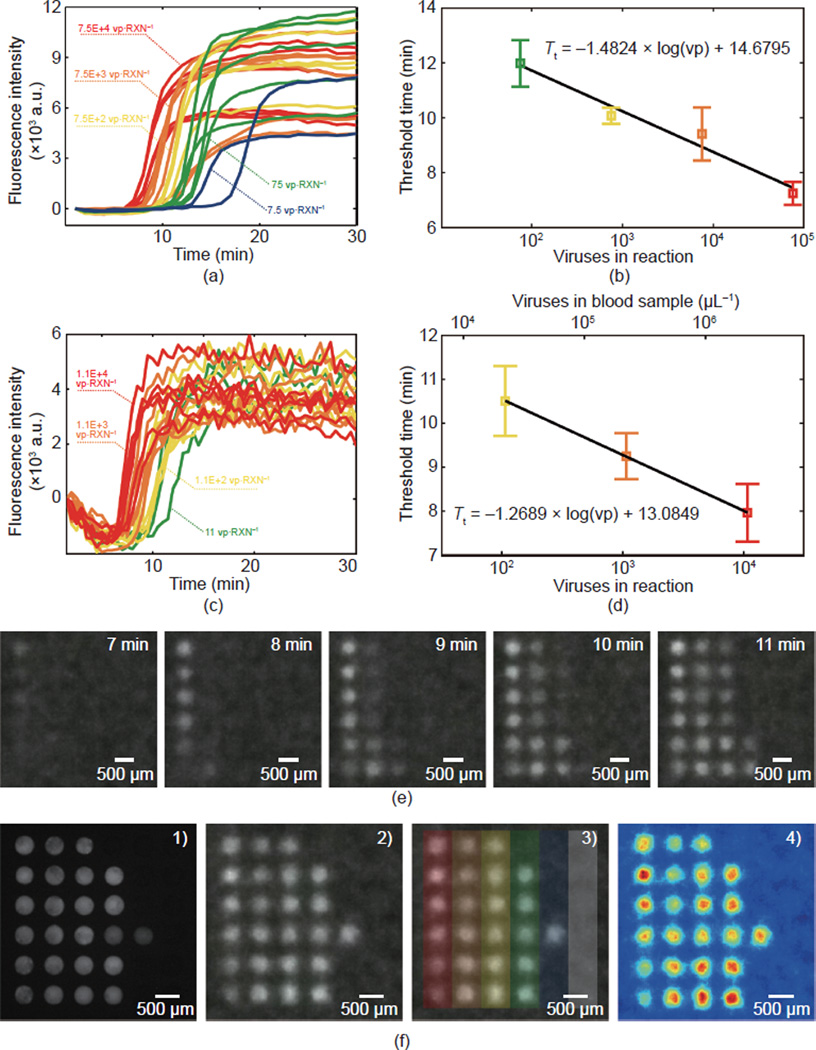
On-chip RT-LAMP for HIV-1 IIIB (a) Baseline-subtracted fluorescence intensity and (b) threshold time versus virus concentration for purified RNA in water on the micro-well substrate imaged with a fluorescence microscope. (c) Baseline-subtracted fluorescence intensity and (d) threshold time versus virus concentration for RNA-spiked lysed whole blood on the micro-well substrate imaged with the smartphone camera. (e) Fluorescence images captured by the smartphone showing the amplification of four RNA concentrations. (f) Endpoint measurements of the same chip in (c)–(e) showing: 1) fluorescence microscopy, 2) the smartphone image, 3) color overlay indicating concentrations (the gray bar indicates negative controls), and 4) a fluorescence intensity colormap created in MATLAB in the process for quantifying the intensity in images.

Next, the RT-LAMP reaction was demonstrated on the microchip. This included experiments with purified RNA in water, shown in Figure 4(a) and (b), and RNA in lysed whole blood (presented in the Supplementary Information), imaged with a fluorescence microscope before the introduction of the smartphone, as shown in Figure 4(c) and (d). This sequence of experiments was performed in order to establish a basis for comparison and to limit the introduction of new variables and sources of noise in each step in the progression of the experiments. All on-chip RT-LAMP measurements were prepared as described in Section 2.4 with DNA LAMP primers pre-spotted and dehydrated on the chip prior to oil immersion, degassing, and reaction droplet placement.

Purified RNA in water was first characterized on the chip. Figure 4(a) displays fluorescence curves measured with a fluorescence microscope, while Figure 4(b) shows the threshold time analysis. In this measurement, data from two droplets were removed due to outlying behavior believed to be due to contamination with inhibitors or experimenter error. This includes one of six reactions in each of the 75 vp· RXN ^−1^ and 7.5E+2 vp·RXN^−1^ samples. Only two of six 7.5 vp·RXN^−1^ samples amplified, and as a result, threshold times for this sample were omitted from the curve in Figure 4(b). In Figure 4(c)–(f), lysed whole blood spiked with viral RNA was imaged with the smartphone apparatus as described in the Section 2.6. Whole virus particles were not used in this measurement, as the apparatus had not yet been converted to be contained within a biosafety cabinet. Figure 4(c) shows fluorescence curves gleaned from the smartphone images, Figure 4(d) shows the threshold time analysis, and Figure 4(e) shows examples of the smartphone fluorescence images every minute for minutes 7–11. The 11 vp·RXN^−1^ (green) and 1.1 v p· RXN ^−1^ (blue) samples are omitted from the threshold time curve because of in-sufficient amplification before 30 min.

Figure 4(f) shows an endpoint measurement obtained following termination of the real-time monitoring at 30 min. Four additional droplets (three of the 11 v p· RXN ^−1^ samples and one 1.1 vp·RXN^−1^ sample) in the array had reacted before the image in Figure 4(f)-1) was captured with the fluorescence microscope. Figure 4(f)-2) is an additional smartphone image captured shortly after the microscopy image was obtained. Figure 4(f)-3) is identical to Figure 4(f)-2) with an additional color-coded overlay that is consistent with the color-concentration convention used throughout this paper. The sixth column on the far right is a negative control containing a blood sample and reaction mix without viral RNA.

Finally, Figure 4(f)-4) is a color map rendering of Figure 4(f)-2) that was produced in MATLAB as an example of the image analysis process.

### 3.4 Compatibility with other viral nucleic acids

Due to the common occurrence of co-infections of HIV and viral hepatitis (HBV and/or HCV), we sought to demonstrate the compatibility of this on-chip RT-LAMP assay with such cases. Because whole virus particles were not available to us for hepatitis viruses, synthetic viral genomes of DNA (HBV) and RNA (HCV) were obtained and tested. Since limited quantities of the synthetic viral genomes were available to us, this experiment was performed in water and not lysed whole blood in order to avoid the ten-fold dilution of nucleic acids that would result from spiking lysed blood with viral nucleic acids and to perform the test at the highest concentration of viral nucleic acids possible.

**Figure 5:**
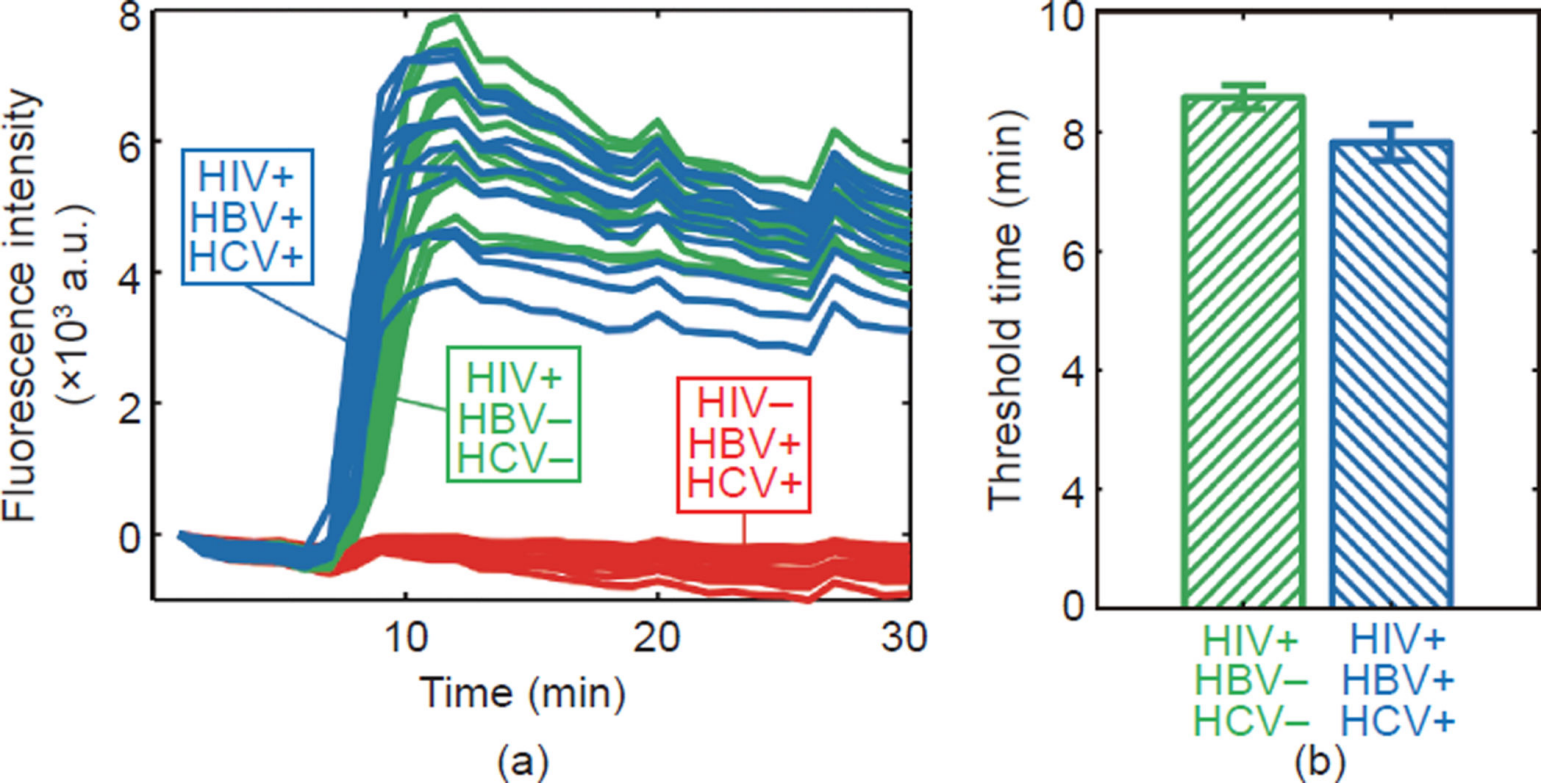
A demonstration of compatibility with common co-infections The HIV-1 on-chip RT-LAMP reaction was tested in the presence of HBV and HCV nucleic acids at concentrations equivalent to 1.6 × 10^3^ of each virus per 60 nL reaction. LAMP primers for HIV-1 detection were dehydrated in each well of the microchip array and purified nucleic acids in water were prepared in various combinations with a primer-less RT-LAMP master mix. The chip was immersed in mineral oil and placed under a fluorescence microscope on a heating stage at 65 °C. (a) Fluorescence measurements from the fluorescence microscopy chip of three combinations: HIV+/HBV−/HCV−, HIV+/HBV+/HCV+, and HIV−/HBV+/HCV+. (b) A bar chart comparing threshold time for HIV RNA-positive samples with and without hepatitis virus nucleic acid present.

Figure 5(a) shows fluorescence data obtained with the fluorescence microscope measurement system for the on-chip RT-LAMP of three samples: HIV both with and without hepatitis virus nucleic acids, and hepatitis virus nucleic acids without HIV. All 24 of the droplets containing HIV RNA successfully amplified (twelve with hepatitis virus nucleic acid, twelve without), regardless of the presence of other nucleic acids. The twelve HIV-negative droplets, all containing hepatitis virus nucleic acids, did not amplify.

Figure 5(b) shows a comparison of threshold time between the two HIV RNA-containing conditions. The average threshold time for HIV-positive droplets that contained hepatitis nucleic acid differed from the droplets that did not by 8.90%. A standard *t* test comparing the two gives a *P* of 1.8718E-6.

### 3.5 Integrated experiments

**Figure 6:**
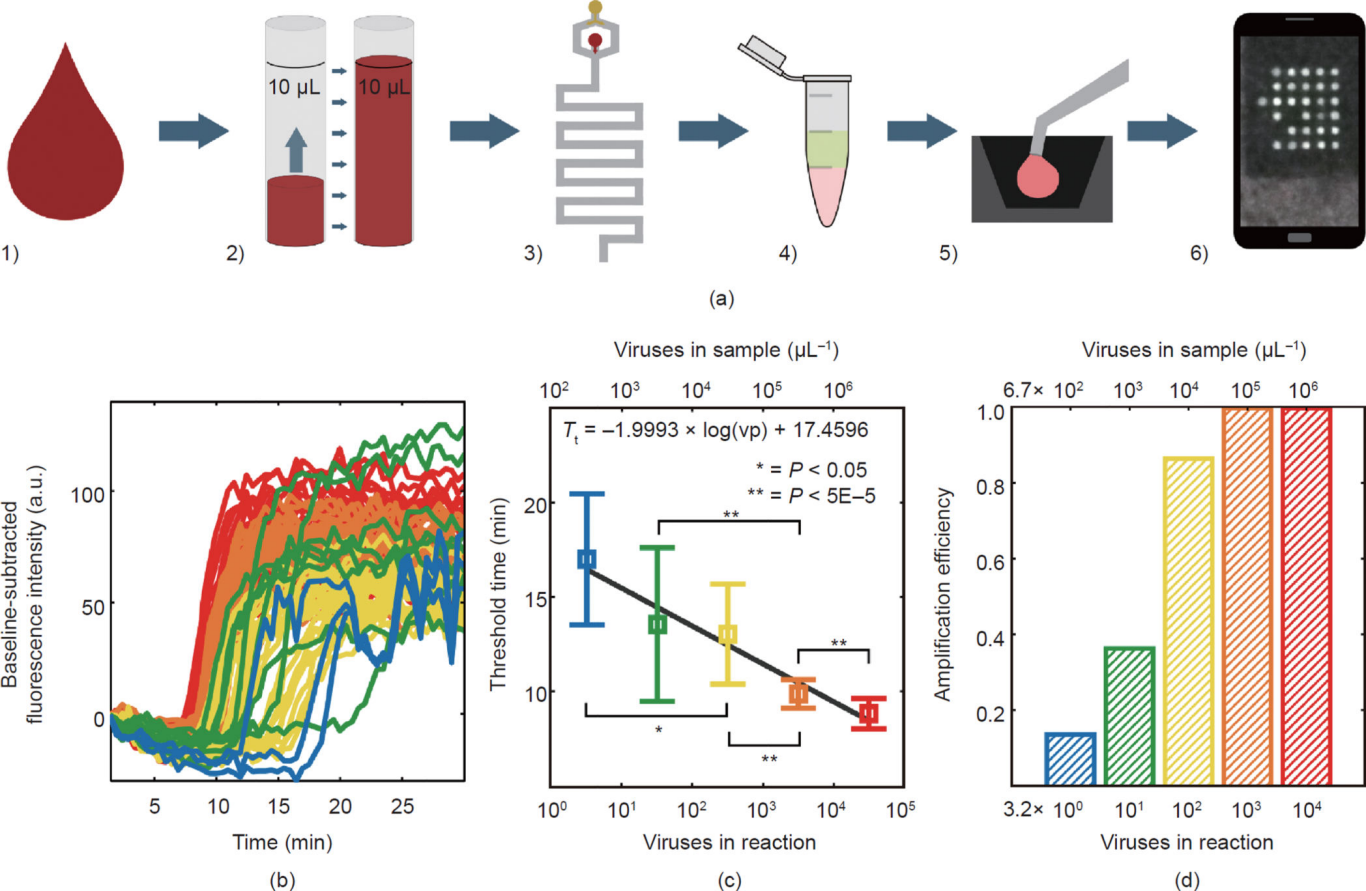
Sample-to-answer RT-LAMP detection of HIV-1 IIIB in lysed whole blood (a) A schematic of the integrated process: 1) Whole blood spiked with HIV-1 IIIB was infused into a microfluidic apparatus; 2) 10 µL of sample was metered based on the volume of the holding coil; 3) the sample was flowed into a microfluidic mixing module at 10 µL·min^−1^ with cell lysis buffer at 40 µL·min^−1^; 4) output from the mixing module was added to the RT-LAMP master mix without primers; 5) a lysed sample with master mix was microinjected onto the microwell substrate prepared with dehydrated primers; 6) the chip was heated to 65 °C in a copper base with a heating stage and the RT-LAMP reaction was monitored by recording a fluorescent image every 30 s using a smartphone. (b) Real-time fluorescence curves as measured by smartphone imaging system. (c) Threshold time values determined by the time at which baseline-subtracted fluorescence intensity exceeded 20% of its maximum value. (d) Amplification efficiency, defined as the fraction of droplets that amplified in the array for each tested concentration.

Integrated experiments were designed to demonstrate the full capacity of this approach for a sample-to-answer solution to point-of-care HIV viral load quantification. Figure 6(a) depicts the complete flow of the process. These experiments differed from the other measurements presented in this paper in that whole blood samples were spiked with whole HIV-1 IIIB virus particles (not viral RNA) at a range of concentrations and each individual sample was analyzed on a separate chip. Of the 36 wells on the microchip array, 6 were used for negative controls and up to 30 were used to test the sample.

Five samples were tested (named A–E), containing approximately 32 000, 3200, 320, 32, and 3.2 whole virus particles per reaction, respectively. Since each reaction droplet contains approximately 4.8 nL of whole blood, this corresponds to viremias in the range of 6.7 × 10^5^–6.7 × 10^9^ viruses per mL of blood, or 1.3 × 10^6^–1.3 × 10^10^ RNA copies per mL of blood plasma (assuming 45% haematocrit).

Due to biosafety considerations, the entire process was adapted, as described previously, to be contained within a biosafety cabinet. This adaptation introduced challenges to the droplet placement, which was performed with a motorized micromanipulator controlled with a joystick and guided by a video feed from a small tabletop microscope. Decreased control over droplet placement led to a decreased success rate in droplet placement. As a result, not all of the 30 wells designated for a virus-positive reaction droplet were used in every measurement. The numbers of successfully placed droplets for samples A through E were as follows: 29, 28, 30, 22, and 22.

Figure 6(b) shows the fluorescence curves, measured with the smartphone system, for all droplets that amplified within 30 min. Figure 6(c) shows the threshold time versus the virus number. The slope of the fit to threshold time versus virus number is −1.9993, which differs in magnitude by 57.6% compared to on-chip RNA in lysed whole blood, by 34.9% compared to on-chip RNA in water, by 18.5% compared to whole virus particles in whole blood in the thermocycler, and by 25.9% compared to purified RNA in water (method 2 in Section 2.1) analyzed in the thermocycler. A *t* test was performed to compare the threshold time and significance was determined by a *P* < 0.05.

In Figure 6(d), we consider a new metric—amplification efficiency— and observe a trend between virus number and the fraction of droplets that amplified. A framework for understanding this phenomenon in the context of digital LAMP measurements is described in the Supplementary Information.

## 4 Discussion

### 4.1 Characterization of RT-LAMP in a benchtop thermocycler

#### Purified viral RNA in water

In the initial thermocycler characterization of this RT-LAMP reaction, we presented a comparison of two methods (see Section 2.1) of viral RNA purification in order to ➀ establish a baseline for a “clean” reaction signal, and ➁ highlight possible factors that may contribute to variations in RT-LAMP results in subsequent analyses.

While the two independent experiments measuring purified RNA in water differed in the manner in which dilutions were performed (before RNA purification in method 1 and after RNA purification in method 2), they also differed in betaine content (0.8 mol· L^−1^ in method 1, 0.4 mol· L^−1^ in method 2). After the initial measurements, betaine concentration was decreased in order to allow for a larger fraction of the reaction volume to consist of sample. One initial hypothesis was that betaine, which contains a cation and reduces secondary-structure formation in DNA, may explain the difference in the two standard curves. However, a careful comparison was performed in which RNA at various low concentrations was added to a common master mix containing 0.4 mol·L^−1^ betaine and, in half of the reactions, additional betaine was added to achieve a concentration of 0.8 mol·L^−1^. The results of this control experiment (see the Supplementary Information) indicated no difference in the threshold time nor in the amplification efficiency at lower concentrations. Therefore, other factors likely explain the vertical offset in the purified RNA experiment in the thermocycler.

We suspect that these factors may include variations in enzyme concentration due to inherent variation in the pipetting process when preparing master mixes, a time-related decrease in enzyme activity (the two experiments were performed several weeks apart and enzyme activity may have decreased with freeze-thaw cycles of the reagent), or variations in ambient temperature and other factors. Additionally, degradation of RNA over time during storage may have decreased the yield of the samples used in method 2, and variations due to manual pipetting may contribute to discrepancies between the actual RNA concentrations gleaned from the two independent purification procedures.

While these factors are important to consider, we chose at this time to acknowledge their potential effects and the need to minimize variation and decided to establish rigorous controls in future experimental or manufacturing processes. Viral RNA stability or yield from the RNA purification process would not affect the results of experiments using whole virus particles. Subsequent analysis involving purified viral RNA or viral RNA spiked in whole blood employed RNA from a third, “fresh” purification identical in protocol to purification method 2. The purified RNA from this process was aliquoted and frozen at −80 °C and thawed only immediately before use in an experiment in order to minimize degradation of the sample between experiments.

#### Comparison with lysed whole blood

To our knowledge, a quantitative RT-LAMP measurement of HIV concentration in whole blood processed only by mixing with a cell lysis buffer has not been described previously in literature. The thermocycler results with whole virus particles spiked in whole blood and mixed with cell lysis buffer suggest the possibility of quantifying virus concentration simply based on reaction kinetics.

The decrease in overall fluorescence intensity indicates that there are fluorescence-quenching components in the lysed whole blood sample. However, because the threshold time is identical when comparing purified RNA and whole blood, we can conclude that whole blood does not affect the amplification efficiency. This is a very promising development, given that a major challenge to most point-of-care diagnostics is the process of isolating analyte from complex biological samples in the absence of controlled environments, skilled technicians, or laboratory instruments [25]. In this case, blood cell lysis is an extremely simple processing step compared to the more complicated techniques described in the literature [6].

### 4.2 Microfluidic blood lysis module

One goal of this paper is to demonstrate the potential for a fully automated RT-LAMP viral load test from a drop (i.e., a finger prick) of whole blood. Such a test requires complete on-chip sample processing from that whole blood drop, which has traditionally been a major barrier for many point-of-care diagnostics approaches. Despite the popularity of PDMS prototyping in this field, many microfluidic and lab-on-a-chip techniques suffer from the ability to translate PDMS devices to commercially viable forms that are compatible with injection molding and other mass-manufacturing techniques [26]. The converse is also true: That designs compatible with manufacturing may be difficult to prototype in PDMS when some properties (e.g., surface-fluid contact angle) are not comparable. Here we demonstrate valve-assisted sample metering and microfluidic mixing resembling a method that we are aware is employed in commercial-grade platforms for blood-sample handling.

Volumetric metering begins with a drop of blood of unspecified size from which 10 µL is precisely metered in a holding coil with an inner diameter of 203.2 µm, reminiscent of commercial microfluidic cartridges that employ volumetric metering. Viable methods have also been employed for fluid mixing in commercial microfluidic designs, making our simple serpentine channel prototyped in PDMS a reasonable design [27].

Data in Figure 3(b) demonstrate that this simple approach of volumetric metering with the serpentine-channel mixing of blood with lysis buffer is compatible with the downstream RT-LAMP analysis. The results from three separate collections show that the method produces an accurate mixing ratio, and that the on-chip mixing is at least as consistent as manual pipetting.

### 4.3 On-chip RT-LAMP

#### Purified RNA with fluorescence microscope imaging

It is unclear what absolute conclusions can be gleaned from a comparison of the fit to the threshold time versus the log of virus number in Figures 2(b) and 4(b). The slope of the fit is smaller in magnitude for the on-chip measurement, suggesting that there may be some chip-related factors leading to a decrease in sensitivity for the quantification of virus number by threshold time analysis. This observation prompted increasing the frequency of images from 60 s (used in thermo-cycler and microscope measurements) to 30 s (used in smart-phone measurements).

The *y*-intercept of the fit in Figure 4(b) is also significantly smaller than those in Figure 2(b) (14.68 min versus 29.1 min and 33.3 min), suggesting that the smaller droplet size may contribute to a more rapid RT-LAMP reaction, a phenomenon that has been discussed elsewhere [28]. This result may be leveraged toward achieving the end goal of a rapid viral load test.

#### Lysed whole blood with smartphone imaging

Data from lysed blood measurements imaged with the smartphone demonstrate two significant steps toward the goals of this paper. The replacement of laboratory hardware (e.g., thermocycler fluorescence detection or fluorescence microscope) with a common smartphone is a core aspect of the novelty of this paper. First, this paper demonstrates that the lysed blood RT-LAMP measurement can be performed with existing mobile technology that is at least as affordable as a high-end smart-phone. Furthermore, trends suggest that mobile communications technologies will continue to improve in capabilities and decrease in cost: an exciting outlook for fluorescence and other optics-based point-of-care diagnostics. Second, our platform (consisting of a 3D printed platform, an LED light source, an emission filter, and a small form-factor heating stage) suggests that, if adequately robust, an add-on component may be developed as an attachment to existing smart-phones, shifting the computation and imaging burden from components integrated with the diagnostic platform to a consumer item that is becoming ubiquitous, even in resource-limited settings [29]. This shift could significantly reduce the production and deployment costs of such a technology.

### 4.4 Compatibility with other viral nucleic acids

Anticipating co-infection with other blood-borne viruses may be important for practical HIV nucleic acid tests, since the incidence of co-infection with HIV and one or more other viruses is high, particularly in populations of intravenous drug users [2, 30]. Most significantly, we have demonstrated that hepatitis viral nucleic acids at high concentrations (equivalent to approximately 1.6 × 10^3^ of each virus per 60 nL reaction) in purified form do not amplify in the RT-LAMP assay that is designed for HIV.

A more detailed analysis gives a *P* of 1.8718E-6 from a standard *t* test, indicating a significant decrease in threshold time for the sample containing nucleic acid from all three viruses versus the sample containing only RNA from HIV. This result indicates a need for more rigorous characterization of this phenomenon in future work. One explanation for a decreased threshold time is the presence of the hepatitis B genome, which is a circular, partially dsDNA, at a relatively high concentration (approximately 1600 copies·RXN^−1^). Its presence may produce an effect that is not seen in whole blood, since mature erythrocytes do not contain DNA, and our simulated co-infected sample would contain a new signal source from dsDNA intercalating dye. Leukocytes in the lysed whole blood sample would be very rare (a few per nL of blood), and genomic DNA from these sources may be packaged and largely inaccessible to the dsDNA dye. Because no amplification was observed in the HIV-negative, HBV/HCV-positive samples, we consider it unlikely that HCV RNA or HBV DNA is acting as a non-specific template for LAMP and producing incorrect reaction products.

### 4.5 Integrated experiments

Our integrated experiments demonstrate the capacity of the test for quantitative viral load measurements based on reaction kinetics or digital statistical methods. The slope of the fit to the threshold time plot in Figure 6(c) is comparable to the original characterization shown in Figure 2, suggesting that the integrated approach has the capacity to be quantitative— and perhaps can be demonstrated to have greater sensitivity upon optimization of reaction chemistry, incubation temperature, and other factors. Bars showing differences that are statistically significant between concentrations indicate the resolution of the integrated test performed here at a 95% confidence level.

Clearly, several factors need to be improved and addressed in order to move toward a fully automated platform. The experiment flow described in Figure 6(a) consists of several components that are compatible with a point-of-care, micro-fluidic cartridge-based *in vitro* diagnostic platform: the volumetric metering of 10 µL of whole blood, microfluidic sample processing, nanolitersized reaction droplets, a silicon chip substrate, and smartphone fluorescence imaging. The process described here does, however, include the manual step of transferring lysed blood from the microfluidic module, mixing it with primer-less RT-LAMP master mix, and placing droplets onto the chip.

We believe the issues requiring this handling step can be addressed by common industry methods not easily demonstrated in the research lab, such as sample distribution into a nanoliter-scale reaction well [31]. The issue of the addition of reaction components to the sample could be addressed by lyophilization or by freeze-drying reagents that are then re-hydrated by the sample. Lyophilized LAMP master mixes have been described in Refs. [32, 33]. In addition, although air-drying reaction components other than primers were problematic on our current platform, we observed that the RT-LAMP master mix used here can be lyophilized in a commercial freeze-drier in 0.2 mL reaction tubes (see the Supplementary Information), and that amplification capabilities are retained. We did not have the equipment available to attempt this process on-chip.

### 4.6 Quantification by threshold time

The measurements presented here exhibit a trend in threshold time versus virus number that suggests that a kinetic measurement based on the time takes an array of droplets to react may be a suitable method to quantify viral load, though the limits of the resolution are still to be determined in an improved platform. For this approach, the sensitivity of the reaction, determined by the slope of the fit to threshold time versus virus number, may need to be increased by optimizing the reaction, including adjustments to enzyme and buffer concentrations or to incubation temperature. Additional optimization would need to be performed in order to improve (i.e., lower) the lower limit of detection of the reaction.

Although these improvements may be made, a lysed whole blood approach will be inherently limited in its capabilities by the reaction volume and dilution factor in lysis buffer. For this reason, a variation on the approach involving digital LAMP may be considered.

### 4.7 Quantification by digital LAMP

Digital LAMP and PCR approaches have been widely described in Refs. [21, 34–39]. The primary advantage of a digital approach is that it relies only on an endpoint measurement—whether a reaction of a small volume amplifies or not—from which concentration can be approximated by measuring hundreds, thousands, or millions of droplets. The approach we describe here could be scaled up for digital LAMP by constructing large arrays of droplets with an automated distribution method.

The upper and lower limits of viral load detection in a digital approach are defined by the number of individual reaction droplets and the total volume of the sample tested. Since the distribution of viruses in reaction droplets is governed by Poisson statistics, we have briefly reviewed these principles in the Supplementary Information for this paper in order to demonstrate the theoretical utility in clinical HIV management of a scaled-up platform that tests a finger-prick droplet of whole blood. For example, a digital LAMP approach requiring just 9 µL of whole blood would be capable of indicating viral loads lower than 500 mL^−1^ of whole blood, with much greater accuracy in the range of 10^4^–10^6^ mL^−1^ (see the Supplementary Information). While this approach cannot compete with the technical specifications of state-of-the-art systems (lower limit < 10 mL^−1^), it would be of practical value, such as in showing declines in viremia following a drug regimen change or in identifying cases of viral rebound in settings where the gold standard of care is inaccessible [40]. The capacity of this platform as a digital LAMP test increases with larger sample sizes and increased number of reaction droplets.

## 5 Conclusions

In performing these experiments and the preparation of this manuscript, the challenge of finding a sample-to-answer point-of-care HIV viral load quantification solution was viewed as two parallel objectives: ➀ sample processing, which traditionally involves isolating or enriching the analyte from its complex matrix, and ➁ analyte detection. We broadly considered various approaches to sample processing that might be integrated with a microchip LAMP approach, many of which were guided by the notion that the presence of cellular material is not compatible with nucleic acid amplification methods. Many of these approaches resulted in the dilution of the analyte by a factor of ten or more, while our approach results in a dilution by only a factor of five, prior to the addition of LAMP reagents with one simple and easily implemented processing step that neither purifies nor enriches. This key merit of our approach can significantly reduce the complexity of and cost for a point-of-care device.

The measurements presented here demonstrate that an RT-LAMP quantification approach is indeed compatible with minimally processed whole blood. To our knowledge, RT-LAMP in lysed whole blood has only been employed by one group, which performed a non-quantitative measurement in a reaction tube on a portable heating device [17]. We demonstrate quantitative detection with the ability to resolve 10-fold changes in concentration above 6.7E+4 µL^−1^ and 100-fold changes in concentrations above 670 µL^−1^. We observed 60 nL droplets with as few as three viruses per reaction amplify, which corresponds to a whole blood virus concentration of 670 µL^−1^. We also discussed that the true power of this approach may be in a quantitative digital LAMP format rather than a kinetic measurement. Our implementation of the lysed whole blood approach in a microchip format with mobile phone imaging represents a significant stride toward a practical solution to viral load measurements in resource-limited settings.

## Supplementary Material

Supplementary Information
