# Supplementary material for: Smartphone-Imaged HIV-1 Reverse-Transcription Loop-Mediated Isothermal Amplification (RT-LAMP) on a Chip from Whole Blood
Source: Engineering (Beijing). Author manuscript; Available in PMC 2015 Dec 22. (PMC4687746; doi:10.15302/J-ENG-2015072)
Supplement: Supplementary Information [file NIHMS742222-supplement-Supplementary_Information.pdf]

Research

Medical Instrumentation—Article

Engineering 2015, 1(3)
DOI 10.15302/J-ENG-2015072

Supplementary Information for
Smartphone-Imaged HIV-1 Reverse-Transcription Loop-Mediated Isothermal Amplification (RT-LAMP) on a Chip from Whole Blood

Gregory L. Damhorst[1,2], Carlos Duarte-Guevara[2,3], Weili Chen[2,3], Tanmay Ghonge[1,2], Brian T. Cunningham[1,2,3], Rashid Bashir[1,2,3]*

1 Schematic of inverted pyramidal microwell structure

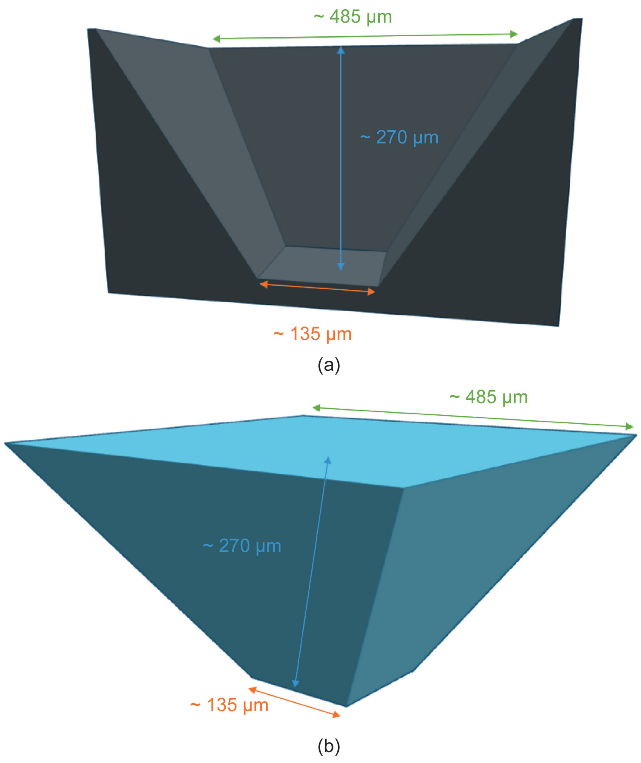

Figure S1. (a) Cross-section of pyramidal well with dimensions indicated; (b) mold of pyramidal well with dimensions indicated.

2 Cell lysis buffer ratio

Methods. For measurements comparing the ratio of blood to lysis buffer, RT-LAMP was performed in the thermocycler with identical mastermixes containing reaction buffers, enzymes, and primers, but where the sample portion consisted of various ratios by volume of whole blood to lysis buffer: 1∶4, 1∶2, 1∶1, or 1∶0. Viruses were spiked into lysed blood following the mixing in order to keep the virus concentration identical between samples.

Results. Figure S2(a) shows threshold time with the 1∶0 case omitted (since it did not exhibit amplification). The results showed differences in threshold time compared to 1∶4 of 0.59% and 1.03% for 1∶2 and 1∶1, respectively, at 670 vp·RXN^{-1} and a difference in threshold time compared to 1∶4 of 0.97% and 3.09% for 1∶2 and 1∶1, respectively, at 67 000 vp·RXN^{-1}. A standard t test produced P compared to 1∶4 for 1∶2 and 1∶1, respectively, of 0.9247 and 0.8444 at 670 vp·RXN^{-1}, and 0.1604 and 0.0138 at 67 000 vp·RXN^{-1}.

Figure S2(b) compares the average maximum baseline-subtracted fluorescence intensity of each condition, demonstrating the trend of increased quenching of fluorescence as absolute blood content increases. In this case, the bar for the 1∶0 (no lysis) condition merely represents the fluctuation of noise, as no amplification was observed in these samples.

Following this characterization, tests were performed to determine if samples prepared at 1∶2 or 1∶1 ratios of blood to lysis buffer could be adequately imaged on-chip with the fluorescence microscope imaging apparatus. This process proved problematic, however, and subsequent lysed blood measurements were performed at the 1∶4 ratio.

Discussion. The characterization of blood-to-lysis buffer ratios confirms that the reduction of fluorescence signal seen in lysed blood versus purified RNA is related to a blood component and not to a component of the lysis buffer, and demonstrates a trend of increasing fluorescence quenching

[1] Department of Bioengineering, The University of Illinois at Urbana-Champaign, Urbana, IL 61801, USA; [2] Micro and Nanotechnology Laboratory, The University of Illinois at Urbana-Champaign, Urbana, IL 61801, USA; [3] Department of Electrical and Computer Engineering, The University of Illinois at Urbana-Champaign, Urbana, IL 61801, USA
* Correspondence author. E-mail: rbashir@illinois.edu
Received 27 July 2015; received in revised form 26 August 2015; accepted 8 September 2015

© The Author(s) 2015. Published by Engineering Sciences Press. This is an open access article under the CC BY license (http://creativecommons.org/licenses/by/4.0/)

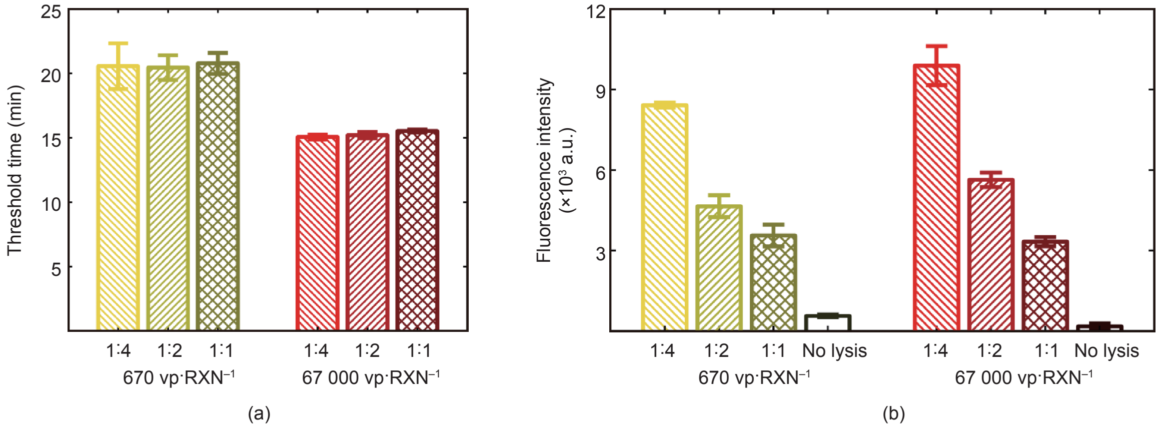

Figure S2. (a) A comparison of threshold time as the ratio of blood to lysis buffer is varied, showing that threshold time does not vary significantly with blood lysis ratio. (b) Plot of the maximum value of baseline-subtracted fluorescence intensity, which decreases as the fraction of blood in the reaction increases. No amplification was observed in un-lysed blood samples.

with increasing blood content. Initially, it was thought that decreasing the volume of lysis buffer relative to the whole blood sample might allow for larger volumes of sample to be employed in the 25 μL or 60 nL reactions, and therefore might increase the detection limit of our platform with respect to the viremia of the original sample. Deviating from the lysis buffer ratio described by Curtis et al. [17], however, proved to be problematic on-chip due to inadequate fluorescence intensity in the microscopy imaging; therefore, we decided to maintain the 1∶4 ratio for subsequent experiments.

The observation that blood concentration affects the fluorescence intensity suggests that one or more components of the lysed blood have a diminishing effect but do not completely quench the fluorescence signal except for when no lysis is performed, while the lack of effect on threshold time suggests that blood components do not interfere with reaction kinetics. This is a critical result in order for quantification of the sample based on reaction kinetics to be possible.

3 Betaine concentration in the RT-LAMP reaction

Initial experiments were performed with 0.8 mol·L^{-1} betaine based on the concentration used by Curtis et al. [17]. However, at a stock concentration of 5 mol·L^{-1}, the betaine reagent occupied 4 μL of the 25 μL reaction—more than any other single reagent. In order to include a greater volume of sample in each reaction, we cut the betaine concentration to 0.4 mol·L^{-1}, a reaction concentration also seen in Ref. [41]. We performed a control experiment to determine if the change in betaine concentration would have any significant effect on the lower limit of detection. Figure S3 shows the raw fluorescence curves from this test, which was performed with low concentrations of purified viral RNA in water. In this test, faster threshold times are observed in 0.4 mol·L^{-1} betaine compared to 0.8 mol·L^{-1} betaine. For the smaller concentration (an average of four viruses per reaction), a third of the reactions amplified in the 0.4 mol·L^{-1} betaine condition while two thirds of the reactions amplified in the 0.8 mol·L^{-1} condition. Although this is not a thorough characterization of the effects of betaine, we considered it adequate indication that the effect of betaine is not significant.

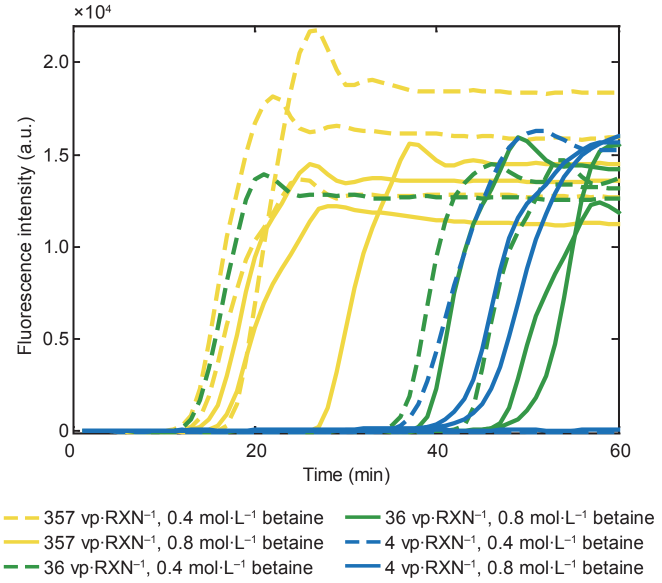

Figure S3. Reaction curves from a comparison of 0.4 mol·L^{-1} and 0.8 mol·L^{-1} betaine in the RT-LAMP reaction.

4 Lysed blood microchip LAMP with microscope imaging

A microchip RT-LAMP experiment with lysed whole blood spiked with viral RNA was performed and imaged with the fluorescence microscope as described in the main text. Figure S4 shows the fluorescence curves and threshold time curve. This experiment was an intermediate step between microchip LAMP with purified RNA in water imaged with a microscope and RNA-spiked lysed whole blood imaged with the smartphone. The data are provided here for thoroughness.

5 Lyophilization of RT-LAMP reagents

To determine whether RT-LAMP reagents could be prepared with a freeze-drying method, we prepared an RT-LAMP mastermix containing buffers, enzymes, primers, and intercalating dye. The mastermix was aliquoted into 0.2 mL reac-

tion tubes and frozen at –20 °C overnight. Four of the frozen reactions were left in the freezer, while eight were kept on ice and transferred to a 2 L Labconco Freeze Dry System. The samples were left in the freeze drier for 5 min after the chamber reached full vacuum, after which the system was vented and the samples were kept slightly below ambient temperature for several hours. Fresh RT-LAMP reactions were then prepared and compared to the frozen mastermixes (kept at –20 °C throughout) and the freeze-dried reagents. Figure S5 depicts the results. Of eight freeze-dried mastermixes, one did not amplify while another showed delayed amplification. Six of the eight reactions amplified in a reasonable time, although threshold time was delayed and less consistent compared to fresh or frozen mastermixes.

We consider this a promising initial test demonstrating the feasibility of freeze-drying RT-LAMP reagents. This protocol was not compatible with on-chip reactions, in which 60 nL droplets evaporated rapidly when exposed to air.

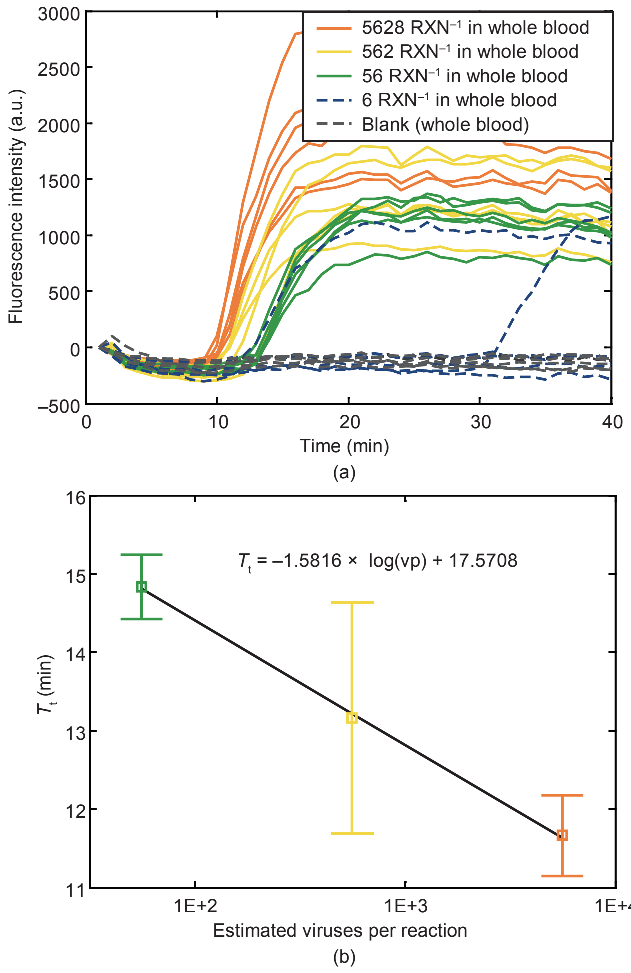

Figure S4. Microchip RT-LAMP of lysed whole blood spiked with RNA viruses. (a) Fluorescence curves; (b) threshold time curve.

6 Statistical model for digital droplet LAMP

The distribution of viruses in small droplets as performed in our method and in any digital PCR or LAMP process is a binomial distribution in which the probability of a "success" is defined by Poisson statistics [36, 42, 43]. The virus concentration from the number of positive droplets in the assay can therefore be calculated using Eq. (S1) [36, 44]:

$$N\lambda = N\ln\left(\frac{N}{N-x}\right) \tag{S1}$$

where N is the total number of droplets; λ is the average number of viruses in a droplet; and x is the number of positive droplets. The concentration of viruses, therefore, is determined by dividing both sides of Eq. (S1) by the volume of the sample, v.

$$c = \frac{N\ln\left(\frac{N}{N-x}\right)}{v} \tag{S2}$$

The uncertainty of this measurement can be determined by calculating a 95% confidence interval (CI) (α = 1.96), the classic form of which is given here [39, 44]:

$$\mathrm{CI} = p \pm \alpha \times \sqrt{\frac{p\times(1-p)}{N}} \tag{S3}$$

where p is the proportion of PCR reaction that amplified (x/N).

However, Shen et al. employed a more sophisticated calculation of confidence intervals based on the so-called "Wilson" method [39]:

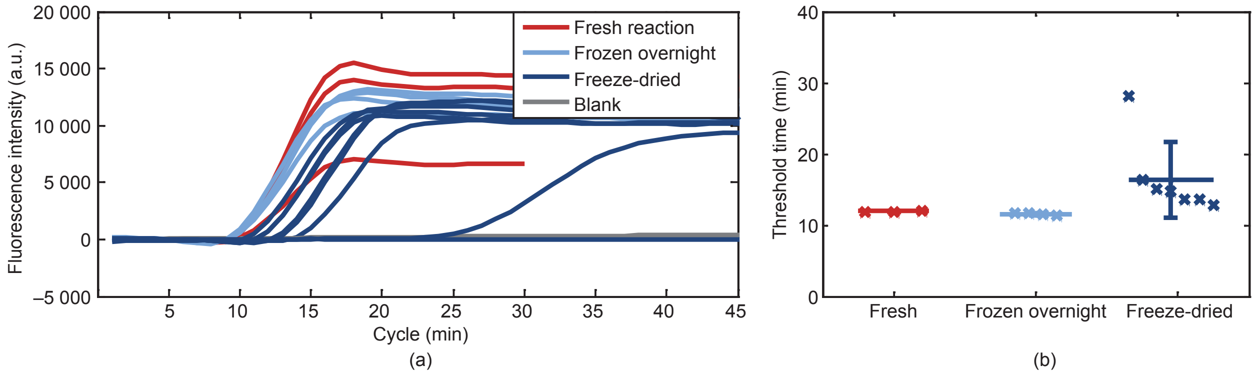

Figure S5. Lyophilization of RT-LAMP mastermix. (a) Fluorescence curves from three different conditions: reactions prepared fresh immediately before testing, reactions for which the mastermix was frozen overnight and kept at –20 °C until immediately before testing, and reactions for which the mastermix was frozen at –20 °C prior to freeze-drying. (b) Threshold times determined by the Eppendorf thermocycler using the CalQ curve-fitting method.

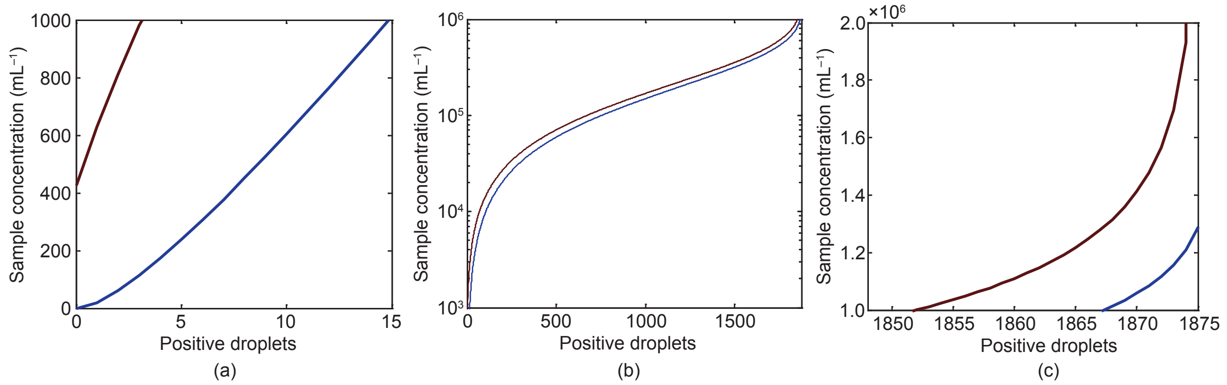

Figure S6. Theoretical limits of a 1875 droplet digital RT-LAMP test utilizing 9 μL of whole blood. Upper and lower bounds determined by a 95% confidence interval are indicated.

$$\mathrm{CI} = \frac{p + \dfrac{\alpha^2}{2\times N} \pm \alpha \times \sqrt{\left[\dfrac{p\times(1-p)}{N} + \dfrac{\alpha^2}{4\times N}\right]\Big/N}}{1+\alpha^2/n} \quad \text{(S4)}$$

To illustrate this method, we will consider a practical finger prick test scenario, where a 10 μL sample is obtained and we assume that 90% of the sample is distributed into our RT-LAMP droplets (60 nL total volume, of which 4.8 nL comes from blood), while 10% is lost to dead volume in the microfluidics.

$$v = 0.9 \times 10\ \mu\mathrm{L} = 9\ \mu\mathrm{L}$$

$$N = \frac{9\ \mu\mathrm{L}}{4.8\ \mathrm{nL}} = 1875$$

As an example, if 19 of the 1875 amplify, Eq. (S4) produces a confidence interval:

$$\left(\frac{x_{\mathrm{high}}}{N}, \frac{x_{\mathrm{low}}}{N}\right) = (0.0065,\ 0.0158)$$

Solving Eq. (S2) for high and low values of x gives us the range of viral loads that this result (19 positive droplets) represents: 1.36×10^3 vp·mL^{-1} to 3.31×10^3 vp·mL^{-1}.

Examining low values for p demonstrates the increasing uncertainty of the digital method as extremes are approached. The 95% confidence interval calculated by the Wilson method indicates an upper limit of 426 mL^{-1} in the case that no droplets amplify in a valid test. One positive droplet of 1875 corresponds to viral loads in the range 20–629 mL^{-1}.

While digital LAMP from a finger prick of blood cannot rival state-of-the-art tests (which have a lower limit of detection of 10 mL^{-1} in plasma and of about 5 mL^{-1} in whole blood), it does offer a working range that would be useful in remote settings where no alternative viral load measurement method is available.

References

1. World Health Organization. HIV/AIDS fact sheet. 2014[2015-08-01]. http://www.who.int/mediacentre/factsheets/fs360/en/#
2. World Health Organization, UNICEF, UNAIDS. *Global Update on HIV Treatment 2013: Results, Impact and Opportunities*. Geneva: WHO Press, 2013
3. J. A. Aberg, J. E. Gallant, K. G. Ghanem, P. Emmanuel, B. S. Zingman, M. A. Horberg; Infectious Diseases Society of America. Primary care guidelines for the management of persons infected with HIV: 2013 update by the HIV medicine association of the Infectious Diseases Society of America. *Clin. Infect. Dis.*, 2014, 58(1): e1–e34
4. Alere. Alere Pima™ CD4. 2012[2015-05-05]. http://alerehiv.com/hiv-monitoring/alere-pima-cd4/
5. Daktari Diagnostics. Products. 2013[2015-05-05]. http://www.daktaridx.com/products/
6. G. L. Damhorst, N. N. Watkins, R. Bashir. Micro- and nanotechnology for HIV/AIDS diagnostics in resource-limited settings. *IEEE Trans. Biomed. Eng.*, 2013, 60(3): 715–726
7. C. F. Rowley. Developments in CD4 and viral load monitoring in resource-limited settings. *Clin. Infect. Dis.*, 2014, 58(3): 407–412
8. US Food and Drug Administration. Complete list of donor screening assays for infectious agents and HIV diagnostic assays. 2013
9. US Food and Drug Administration. Vaccines, blood & biologics: HIV-1. 2010[2014-03-17]. http://www.fda.gov/BiologicsBloodVaccines/BloodBloodProducts/ApprovedProducts/LicensedProductsBLAs/BloodDonorScreening/InfectiousDisease/ucm126582.htm
10. T. Peterson, M. Stuart. HIV Testing Overview. 2011 [2014-03-17]. http://emedicine.medscape.com/article/1983649-overview
11. X. Zhang, S. B. Lowe, J. J. Gooding. Brief review of monitoring methods for loop-mediated isothermal amplification (LAMP). *Biosens. Bioelectron.*, 2014, 61: 491–499
12. T. Notomi, et al. Loop-mediated isothermal amplification of DNA. *Nucleic Acids Res.*, 2000, 28(12): e63
13. M. P. de Baar, E. C. Timmermans, M. Bakker, E. de Rooij, B. van Gemen, J. Goudsmit. One-tube real-time isothermal amplification assay to identify and distinguish human immunodeficiency virus type 1 subtypes A, B, and C and circulating recombinant forms AE and AG. *J. Clin. Microbiol.*, 2001, 39(5): 1895–1902
14. M. P. de Baar, et al. Single rapid real-time monitored isothermal RNA amplification assay for quantification of human immunodeficiency virus type 1 isolates from groups M, N, and O. *J. Clin. Microbiol.*, 2001, 39(4): 1378–1384
15. K. A. Curtis, D. L. Rudolph, S. M. Owen. Rapid detection of HIV-1 by reverse-transcription, loop-mediated isothermal amplification (RT-LAMP). *J. Virol. Methods*, 2008, 151(2): 264–270
16. C. Liu, et al. An isothermal amplification reactor with an integrated isolation membrane for point-of-care detection of infectious diseases. *Analyst (Lond.)*, 2011, 136(10): 2069–2076
17. K. A. Curtis, et al. Isothermal amplification using a chemical heating device for point-of-care detection of HIV-1. *PLoS ONE*, 2012, 7(2): e31432
18. K. A. Curtis, P. L. Niedzwiedz, A. S. Youngpairoj, D. L. Rudolph, S. M. Owen. Real-time detection of HIV-2 by reverse transcription-loop-mediat-

ed isothermal amplification. *J. Clin. Microbiol.*, 2014, 52(7): 2674–2676
19. C. Liu, et al. Membrane-based, sedimentation-assisted plasma separator for point-of-care applications. *Anal. Chem.*, 2013, 85(21): 10463–10470
20. F. B. Myers, R. H. Henrikson, J. M. Bone, L. P. Lee. A handheld point-of-care genomic diagnostic system. *PLoS ONE*, 2013, 8(8): e70266
21. B. Sun, F. Shen, S. E. McCalla, J. E. Kreutz, M. A. Karymov, R. F. Ismagilov. Mechanistic evaluation of the pros and cons of digital RT-LAMP for HIV-1 viral load quantification on a microfluidic device and improved efficiency via a two-step digital protocol. *Anal. Chem.*, 2013, 85(3): 1540–1546
22. N. N. Watkins, et al. Microfluidic CD4+ and CD8+ T lymphocyte counters for point-of-care HIV diagnostics using whole blood. *Sci. Transl. Med.*, 2013, 5(214): 214ra170
23. C. Duarte, E. Salm, B. Dorvel, B. Reddy Jr., R. Bashir. On-chip parallel detection of foodborne pathogens using loop-mediated isothermal amplification. *Biomed. Microdevices*, 2013, 15(5): 821–830
24. P. Khlebovich. IP Webcam. 2015
25. G. L. Damhorst, M. Murtagh, W. R. Rodriguez, R. Bashir. Microfluidics and nanotechnology for detection of global infectious diseases. *P. IEEE*, 2015, 103(2): 150–160
26. G. Jenkins, C. D. Mansfield. *Microfluidic Diagnostics: Methods and Protocols*. New York: Humana Press, 2013
27. C. D. Chin, V. Linder, S. K. Sia. Commercialization of microfluidic point-of-care diagnostic devices. *Lab Chip*, 2012, 12(12): 2118–2134
28. S. Y. Teh, R. Lin, L. H. Hung, A. P. Lee. Droplet microfluidics. *Lab Chip*, 2008, 8(2): 198–220
29. The World Bank. Mobile phone access reaches three quarters of planet's population. 2012[2015-05-22]. http://www.worldbank.org/en/news/press-release/2012/07/17/mobile-phone-access-reaches-three-quarters-planets-population
30. A. S. F. Lok, B. J. McMahon. Chronic hepatitis B: Update 2009. *Hepatology*, 2009, 50(3): 661–662
31. M. Baker. Digital PCR hits its stride. *Nat. Methods*, 2012, 9(6): 541–544
32. Y. Chander, et al. A novel thermostable polymerase for RNA and DNA loop-mediated isothermal amplification (LAMP). *Front. Microbiol.*, 2014, 5: 395
33. C. C. Boehme, et al. Operational feasibility of using loop-mediated isothermal amplification for diagnosis of pulmonary tuberculosis in microscopy centers of developing countries. *J. Clin. Microbiol.*, 2007, 45(6): 1936–1940
34. A. C. Hatch, et al. 1-Million droplet array with wide-field fluorescence imaging for digital PCR. *Lab Chip*, 2011, 11(22): 3838–3845
35. R. H. Sedlak, K. R. Jerome. Viral diagnostics in the era of digital polymerase chain reaction. *Diagn. Microbiol. Infect. Dis.*, 2013, 75(1): 1–4
36. K. A. Heyries, et al. Megapixel digital PCR. *Nat. Methods*, 2011, 8(8): 649–651
37. C. M. Hindson, et al. Absolute quantification by droplet digital PCR versus analog real-time PCR. *Nat. Methods*, 2013, 10(10): 1003–1005
38. R. A. White III, S. R. Quake, K. Curr. Digital PCR provides absolute quantitation of viral load for an occult RNA virus. *J. Virol. Methods*, 2012, 179(1): 45–50
39. F. Shen, W. Du, J. E. Kreutz, A. Fok, R. F. Ismagilov. Digital PCR on a SlipChip. *Lab Chip*, 2010, 10(20): 2666–2672
40. M. Pai, M. Ghiasi, N. P. Pai. Point-of-care diagnostic testing in global health: What is the point? *Microbe*, 2015, 10(3): 103–107
41. K. A. Curtis, D. L. Rudolph, S. M. Owen. Sequence-specific detection method for reverse transcription, loop-mediated isothermal amplification of HIV-1. *J. Med. Virol.*, 2009, 81(6): 966–972
42. J. E. Kreutz, T. Munson, T. Huynh, F. Shen, W. Du, R. F. Ismagilov. Theoretical design and analysis of multivolume digital assays with wide dynamic range validated experimentally with microfluidic digital PCR. *Anal. Chem.*, 2011, 83(21): 8158–8168
43. R. Luo, M. J. Piovoso, R. Zurakowski. Modeling uncertainty in single-copy assays for HIV. *J. Clin. Microbiol.*, 2012, 50(10): 3381–3382
44. R. Haynes. Principles of digital PCR and measurement issues: The certification of Cytomegalovirus Standard Reference Material (SRM 2366) as a model for future SRMs. In: *Digital PCR Applications and Advances*. San Diego, CA, USA, 2012
